# Chronic stress and body condition of wolf-killed prey in Prince Albert National Park, Saskatchewan

**DOI:** 10.1093/conphys/coz037

**Published:** 2019-07-10

**Authors:** Justin R Shave, Andrew E Derocher, Seth G Cherry, Gregory W Thiemann

**Affiliations:** 1 Department of Biological Sciences, University of Alberta, Edmonton, Canada; 2 Parks Canada Agency, Radium Hot Springs, Canada; 3 Faculty of Environmental Studies, York University, Toronto, Ontario, Canada

**Keywords:** Bison, cortisol, gray wolf, marrow fat, predation, stress

## Abstract

Chronic stress and poor body condition can cause adverse physiological and behavioural responses and may make animals more vulnerable to predation. We examined hair cortisol concentration (HCC) and marrow lipid content, as bioindicators of chronic stress and body condition, respectively, of bison (*Bison bison bison*), moose (*Alces alces*) and white-tailed deer (*Odocoileus virginianus*) killed by wolves (*Canis lupus*) in Prince Albert National Park (PANP), Saskatchewan, Canada. The Sturgeon River plains bison population in PANP is one of only a few wild populations of plains bison in their historical range in Canada and has experienced a decline of around 50% since 2005. We expected wolf-killed bison to have elevated HCC compared to human-harvested bison and that there would be a negative relationship between HCC and marrow lipids among wolf-killed animals. We compared HCC between different mortality sources for bison (wolf-killed *n* = 20 or human-harvested *n =* 23) and found that HCC was significantly elevated in wolf-killed bison (¯ = 7.56 ± 1.35 pg/mg). We found that HCC, species, sex and snow depth were all significant predictor variables of marrow lipid content of bison (*n* = 14), moose (*n* = 11) and deer (*n* = 27). Bison displayed the strongest negative correlation between HCC and marrow lipid content (r^2^ = 0.31). Our results suggest that chronic stress and poor body condition make prey more vulnerable to predation by wolves. HCC and marrow lipid content can provide reliable indicators of the physiological response of animals to stressors and may provide information on expected predator success that can be used to predict predator population dynamics.

## Teaser

Chronic stress and poor body condition may make animals vulnerable to predation. We examined cortisol and marrow lipids as bioindicators of stress and condition, respectively, in wolf-killed ungulates. We found that wolf-killed bison had elevated cortisol, and there was a negative correlation between cortisol and marrow lipids.

## Introduction

In vertebrates, adverse stressors cause the release of glucocorticoids (GCs) from the adrenal cortex via the hypothalamic–pituitary–adrenal (HPA) axis (Creel *et al.*, 2009). The release of GC is a component of the stress response and plays a key role in allostasis, which is the active process of maintaining and/or reestablishing homeostasis (McEwen, 1998). GCs, such as cortisol, cause behavioural and/or physiological responses that form an adaptive stress response (Le *et al.*, 2005). Stressors that stimulate GC release in wildlife can include parasitism, disturbance, reduction in habitat quality, predation, human hunting pressure and climate change (Kunkel and Pletscher, 2000; Chapman *et al.*, 2006; Lankester, 2010; Wasser *et al.*, 2011; Jaimez *et al.*, 2012; Mislan *et al.*, 2016). Acute stress is an immediate adaptive response to adverse stimuli with short-term physiological effects, but chronic or repeated activation of the stress response can be maladaptive and negatively affect growth, reproduction and cognitive ability, increase catabolism of stored energy and depress immune activity (Boonstra *et al.*, 1998; Sapolsky *et al.*, 2000; Kitaysky *et al.*, 2003; Charmandari *et al.*, 2005; Macbeth *et al.*, 2010).

Measurement of circulating GC in wildlife can be invasive (e.g. capture and restraint), which can initiate the stress response and alter GC levels (Kersey and Dehnhard, 2014). Minimally invasive methods of GC quantification often use feces, urine, feathers and hair as alternatives (Lafferty *et al.*, 2015; Carlsson *et al.*, 2016; Dantzer *et al.*, 2016; Di Francesco *et al.*, 2017; Seeber *et al.*, 2018). Quantification of fecal GCs has been conducted on a wide range of taxa and provides a measure of stress during the time of gut passage (Bonier *et al.*, 2004; Schwarzenberger, 2007; Kersey and Dehnhard, 2014; Rolland *et al.*, 2017). Cortisol is incorporated into hair during growth, and hair cortisol concentration (HCC) is advantageous for examining chronic stress as it provides a long-term record of stress (e.g. weeks to months) without the need for repeated sampling (Sheriff *et al.*, 2011; Meyer and Novak, 2012). HCC has been analyzed in numerous mammals, but fewer studies examine the relationship between HCC and body condition (Mislan *et al.*, 2016; Di Francesco *et al.*, 2017; Ewacha *et al.*, 2017; Heimbürge *et al.*, 2019).

Body condition can be assessed using a variety of morphological or physiological indices, including internal or external fat repositories and measurements, such as mass or size (Cattet, 1990; Stephenson *et al.*, 1998; Labocha and Hayes, 2012; Risco *et al.*, 2018). Another commonly used method to assess body condition in large mammals is marrow lipids (Neiland, 1970; Mech, 2007; Yamanaka *et al.*, 2011; Borowik *et al.*, 2016). Marrow lipids are the last energy stores to be metabolized in times of food shortage, and low marrow lipid percentage (10–30%) is indicative of poor body condition (Cheatum, 1949; Raglus *et al.*, 2019). The marrow core in nutritionally stressed individuals appears gelatinous and/or red or yellow due to higher water content, low red blood cell formation and anemia (Cheatum, 1949). Poor body condition can result from low food quality, reproduction, parasitism and environmental factors such as winter severity (Delgiudice *et al.*, 2001; Tollefson *et al.*, 2010; Debeffe *et al.*, 2016; Gardner *et al.*, 2016). In turn, prey body condition, in addition to age, sex and encounter rates, can influence vulnerability to predation (Huggard, 1993b, 1993c). Animals killed by predation may have less marrow fat than the same species killed by car accidents (Mech, 2007) or human-harvests (Sand *et al.*, 2012).

Understanding the relationship between ungulate stress response and body condition, using HCC and marrow lipid content as bioindicators, respectively, can be useful for assessing environmental stressors affecting population status. Negative correlations between GC concentrations, body condition and immunocompetence are common across taxa (Boonstra, 2004; Rich and Romero, 2005; Charbonnel *et al.*, 2007; Mislan *et al.*, 2016). Consequently, the combined effect of low reproductive output, poor body condition and reduced immunocompetence of individuals in a population, as a result of chronic stress, may impact population viability (Boonstra *et al.*, 1998; Mumby *et al.*, 2015).

We evaluated the link between chronic stress, body condition and vulnerability to predation by gray wolves (*Canis lupus*) in bison (*Bison bison bison*), moose (*Alces alces*) and white-tailed deer (*Odocoileus virginianus*) in Prince Albert National Park (PANP), Saskatchewan, Canada. The Sturgeon River plains bison (SRPB) population in PANP is one of only a few wild populations of plains bison in their historical range in Canada and has experienced a decline of around 50% since 2005 (Merkle *et al.*, 2015). Therefore, we examined how stress and body condition may be increasing bison vulnerability to predation. We used prey HCC and marrow lipids as bioindicators of chronic stress and body condition, respectively. We hypothesized that chronic stress levels would differ based on the cause of mortality. We tested this hypothesis by comparing HCC collected from human-harvested versus wolf-killed bison. We predicted that wolf-killed bison would have elevated HCC, as chronic stress would increase vulnerability of bison to predation. We also hypothesized that HCC would predict body condition of ungulates killed by wolves, when accounting for environmental factors and age class of prey. We predicted that there would be a negative correlation between HCC and marrow lipid content in wolf-killed ungulates.

## Methods

### Study area

**Figure 1:**
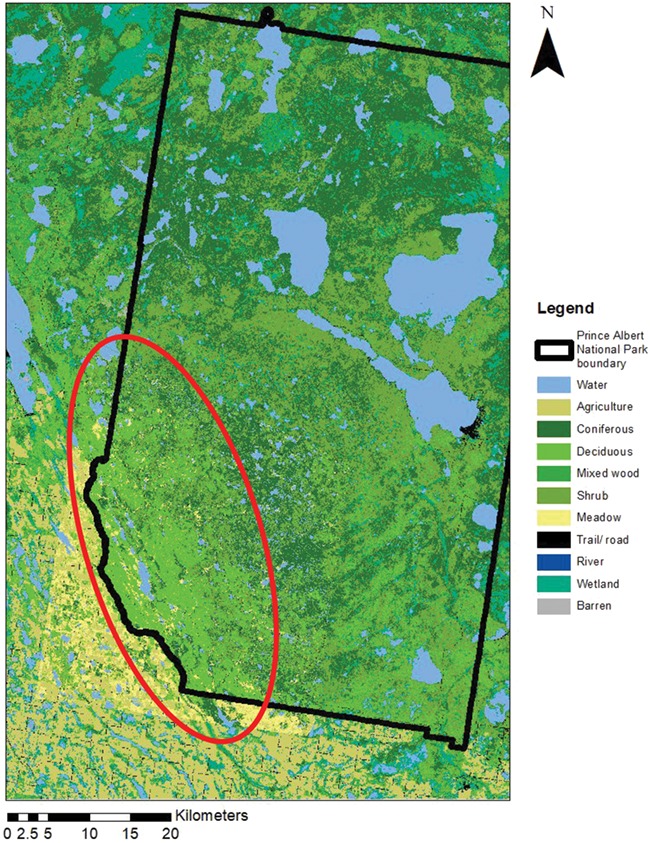
Map of PANP, Saskatchewan with our study area indicated by the red ellipse.

Our study area was in the southwest corner of PANP (centered at 53.7246° N, 106.6754° W; Fig. 1). This area is characterized by aspen parkland and remnant native fescue grassland in the south, boreal forest in the north and agricultural land outside of PANP to the west. The upland areas are dominated by trembling aspen (*Populus tremuloides*), white spruce (*Picea glauca*) and jack pine (*Pinus banksiana*), and the lowland areas are dominated by black spruce (*Picea mariana*) and larch (*Larix laracina*; Fortin *et al.,* 2002). The area has long, cold winters (daily mean temperature for January: −16.5°C) and short, warm summers (daily mean temperature for July: +17.7°C) and receives an average of 450 mm of annual precipitation, the majority of which falls as rain.

### Sample collection

We used wolf Global Positioning System-collar data on two wolf packs to track and identify kill sites where bison (*n* = 20), moose (*n* = 11) and deer (*n* = 27) samples were collected. We identified kill sites using a rule-based algorithm programmed in Python TM language (Python Software, Hampton, NH, USA; Knopff *et al.*, 2009) that identified clusters of GPS points that were geographically within 300 m and temporally within 4 days of each other (Sand *et al.*, 2005). Kill sites were visited in November to March from 2013 to 2017. We used skeletal or hair remains from prey carcasses to identify species and sex–age class (young of year, yearling or adult). Remains of prey that we could not sex in the field were sexed using DNA extraction and single-locus analysis at Wildlife Genetics International (Nelson, BC, Canada) from ~50 mg of muscle sample. A 10–12 cm section from the middle of the femur was cut using a handsaw for marrow lipid analysis. Hair samples for cortisol analysis were collected by plucking from the prey carcass or from clumps of hair on the ground at the kill site.

Hair samples from bison (*n* = 23) were also collected during legal harvesting events that occurred outside of PANP (Bergeson, 1993). At the harvest location, we collected hair and muscle tissue samples and determined the age and sex of bison using the horns and teeth (Fuller, 1959). We collected samples during late summer to late autumn from 2013 to 2015, when most harvests occur as bison are more likely to leave PANP to graze on agronomic plants (Sigaud *et al.*, 2017). All samples were stored frozen (−20°C) until analysis.

### Marrow lipid analysis

We determined marrow lipid content for animals killed by wolves by scooping marrow out of a cross-sectional cut and discarding any exposed surface, which may have been subject to oxidation during storage. We quantitatively extracted the marrow lipid using a modified Folch extraction (Folch *et al.*, 1957; Iverson *et al.*, 2001), with a 2:1 chloroform-methanol (Chl:MeOH) solution and 0.1 mg/ml butylated hydroxytoluene to prevent oxidation. Marrow lipid content was expressed as the percent of total sample wet weight.

### HCC analysis

We used guard hairs for cortisol analysis as there is less variation among and within body regions in comparison to underfur (Macbeth *et al.*, 2010). Bison, moose and deer undergo an annual moult in the spring, after which the winter coat of underfur and overlying guard hair is grown over the summer to late autumn (Peterson, 1978; Meagher, 1986). Guard hairs reflect cortisol levels during the summer and autumn before sample collection, as cortisol is integrated during hair growth. Surface contaminants on hair (e.g. blood, urine, feces, etc.) may contain cortisol and can confound HCC analysis (Macbeth *et al.*, 2010; Cattet *et al.*, 2014). We removed surface contaminants from hair by performing two 3-min methanol washes (0.1 ml of methanol/mg hair) per sample and drying the samples underneath a fume hood for 2 days. Hair was ground to a fine powder, and HCC was extracted and analyzed at the Veterinary Biomedical Centre (University of Saskatchewan, Saskatoon, SK, Canada) following Macbeth *et al.* (2010). We conducted assay validation on bison samples, as this is the first record (that we know of) of HCC analysis for this species. The cortisol extraction efficiency was >95% (Macbeth *et al.*, 2010). Parallelism between serially diluted bison hair extracts and standard cortisol concentrations was observed (R^2^ = 0.994, *P* = 0.19). The intra-assay coefficient of variability (*n* = 6) was 9.36% and the inter-assay coefficient of variability (*n* = 12) was 11.78%.

**Figure 2:**
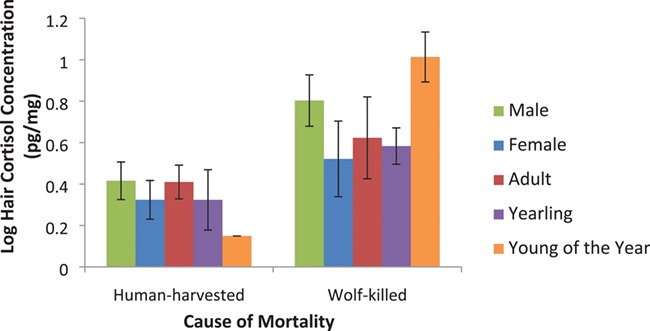
Comparison of HCCs between human-harvested (*n* = 23) and wolf-killed (*n* = 20) plains bison. Mean values (± SE) are shown for each group. Samples from wolf-killed bison were collected at wolf kill sites visited from November to March in 2013 to 2017 in the SW corner of PANP, Saskatchewan. Samples from human-harvested bison were collected from harvest events that occurred outside of the park from August to November in 2013 to 2016

### HCCs of wolf-killed versus human-harvested bison

We examined the difference between HCC (log-transformed) of wolf-killed and human-harvested bison. We tested assumptions of normality and homogeneity of variance of our transformed data, and we used a *t*-test to examine the HCC of wolf-killed or human-harvested bison if assumptions were not violated. We compared a series of linear mixed effects models to determine which factors best predicted HCC in bison. Variables in our models included mortality source (wolf-killed or human-harvested), age (young of year, yearling and adult) and sex of bison. We included an interaction term between age and sex, and year as a random effect. Because all of our models were nested, we used likelihood-ratio tests (LRTs) to examine whether the more complex model was justified by the reduction in likelihood.

### Factors affecting prey body condition

We examined the relationship between marrow lipid content and HCC in prey samples from wolf kill sites. We used generalized linear mixed effects models to examine the relationship, using a binomial family for our models and a log-odds link function. We included the variables HCC, species, sex, mean snow depth from the 2 weeks before sample collection and mean low temperature from the 2 weeks before sample collection. We included year and site as a random effect, to account for variation between years and wolf packs. We used Spearman’s test of correlation to determine and exclude highly correlated variables (r^2^ > 0.60). Since all of our models were nested, we used LRTs to examine whether the more complex model was justified by the reduction in likelihood and accounted for the error associated with multiple comparisons using a Bonferroni correction (Bland and Altman, 1995; Cabin and Mitchell, 2000). All analyses were conducted in R (R Core Team, 2017) using the package lme4 (Bates *et al.*, 2014) with an alpha (α) value of 0.05.

## Results

### HCCs of wolf-killed versus human-harvested bison

The transformed data followed a normal distribution (Shapiro–Wilk test: W = 0.96, *P* > 0.05) and had equal variance across samples (Bartlett test: K^2^ = 1.31, *P* > 0.05), so we used a *t*-test to compare HCC of wolf-killed (*n* = 20) to harvested bison (*n* = 23). HCC was significantly higher in wolf-killed bison (Fig. 2; two-sample *t*-test: t = 8.36, *P* < 0.001). We constructed four different models of varying complexity to explain HCC, including the null model (Table 1). Source of mortality (human-harvested versus wolf-killed) was the best predictor of HCC in bison (LRT: D = 7.86, *P* < 0.01; Table 2). Males had elevated HCC in both harvested and wolf-killed bison, but sex and age variables were not significant.

**Table 1:** Linear mixed effects models used to investigate factors affecting HCC for plains bison in PANP, Saskatchewan

Model number	Model description
1	HCC ~ 1+ (1|year)
2	HCC ~ mortality source + (1|year)
3	HCC ~ mortality source + age + (1|year)
4	HCC ~ mortality source + age + sex + (1|year)
5	HCC ~ mortality source + age + sex + age:sex + (1|year)

Variables included in the most complex model are mortality source (human-harvested or wolf-killed), age, sex and an interaction between age and sex, with the simplest model being the null model. All models included a random effect of year.

**Table 2:** LRTs of linear mixed effects models used to investigate the relationship between HCC and mortality source, age and sex of plains bison in PANP, Saskatchewan, using an alpha (α) = 0.05

Models compared	D	df	*P*-value
1 vs 2	7.86	1	<0.01
2 vs 3	1.26	1	>0.05
3 vs 4	0.08	1	>0.05
4 vs 5	0.95	1	>0.05

If *P* < α then the more complex model was justified by the reduction in likelihood.

**Table 3:** Ranges of HCCs and marrow lipids measured from hair and femur samples, respectively, from wolf-killed bison, deer and moose collected during the winter from 2013 to 2017 in PANP, Saskatchewan

Species	Hair cortisol concentration (Mean ¯ pg/mg ± SE)	Marrow lipid content (Mean ¯ % ± SE)
Bison	6.15 ± 1.46	74.57 ± 6.34
Deer	6.81 ± 0.48	77.22 ± 4.09
Moose	9.11 ± 1.25	55.27 ± 9.05

**Table 4:** Generalized linear mixed effects models used to investigate factors affecting body condition for bison, moose and deer killed by wolves in PANP, Saskatchewan

Model number	Model description
1	% Marrow lipid ~ 1 + (1|site) + (1|year)
2	% Marrow lipid ~ HCC + (1|site) + (1|year)
3	% Marrow lipid ~ HCC + species + (1|site) + (1|year)
4	% Marrow lipid ~ HCC + species + sex + (1|site) + (1|year)
5	% Marrow lipid ~ HCC + species + sex + snow depth + (1|site) + (1|year)

Variables included in the most complex model are HCC, species, sex and snow depth, with the simplest model being the null model. All models included a random effect of site (i.e. wolf pack) and year.

**Table 5:** LRTs of generalized linear mixed effects models used to investigate the relationship between marrow lipid content and HCC of bison, moose and deer in PANP, Saskatchewan, using an alpha (α) = 0.05

Models compared	D	df	*P*-value
1 vs 2	53.40	1	<0.001
2 vs 3	22.35	1	<0.001
3 vs 4	62.77	1	<0.001
4 vs 5	28.53	1	<0.001

If *P* < α then the more complex model was justified by the reduction in likelihood.

### Factors affecting prey body condition

**Figure 3:**
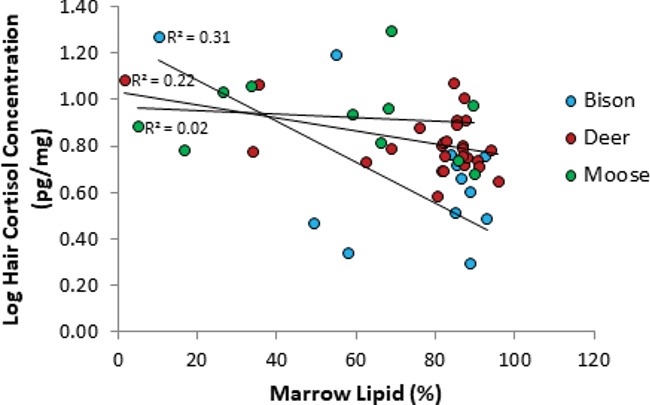
Linear regressions examining the relationship between marrow lipid percentage and HCC for wolf-killed bison (*n* = 14), moose (*n* = 11) and deer (*n* = 27). Hair and bone marrow samples were collected from wolf kill sites visited from November to March in 2013 to 2017 in PANP, Saskatchewan

HCC and marrow lipids ranged from ¯ = 6.2 ± 1.5 to 9.1 ± 1.3 pg/mg and ¯ = 55.3 ± 9.1 to 77.2 ± 4.1%, respectively, between species (Table 3). We constructed five models of varying complexity to explain marrow lipids, including the null model, and we included site and year as random effects to account for variation between wolf packs and years (Table 4). Mean snow depth and mean minimum temperature were negatively correlated (Spearman’s correlation: r^2^ = −0.64, S = 38 535, *P* < 0.001), so we used snow depth as a measure of winter severity. We found that the addition of all variables (HCC, species, sex and snow depth) significantly added to the fit of the models, with species explaining the least in regards to marrow lipid content (Table 5). Bison displayed the strongest negative correlation between HCC and marrow lipid percentage (Pearson correlation: r^2^ = 0.31; Fig. 3). Females had on average higher marrow lipid content than males, and marrow lipid content decreased with increasing snow depth.

## Discussion

Wolf-killed bison had significantly higher HCC than human-harvested bison, and mortality source was the best predictor of HCC in bison. The range of HCC in bison is comparable to other taxa (Di Francesco *et al.*, 2017). There is considerable inter-individual variation in HCC within a species, which may indicate the health status of individuals (Lafferty *et al.*, 2015; Caslini *et al.*, 2016). Because most hair samples were collected from wolf-killed animals, we were unable to sample the same body region on each individual, as it was dependent on prey remains. HCC can vary between body regions (Sharpley *et al.*, 2010; Terwissen *et al.*, 2013; Carlitz *et al.*, 2015), but we would expect our range of HCC to be larger if this was biasing our results. Marrow lipid analysis provided information over the autumn and winter, based on the period of sample collection, whereas HCC provided measurement of stress over the summer and autumn, based on hair growth in ungulates. While the measurement period of each bioindicator differed, both quantify stress and body condition over weeks to months. Ideally, stress and body condition would be analyzed concurrently to better examine a finer scale relationship between the two variables.

Chronic stress can result in adverse physiological responses, and individuals with high circulating GC concentrations may experience muscle wasting, immunosuppression, a decrease in reproductive ability, growth suppression and reduced body condition (Boonstra and Singleton, 1993; Boonstra *et al.*, 1998; Charbonnel *et al.*, 2007). However, few studies have identified the effects of chronic stress on the fitness of free-ranging animals, and high levels of circulating GC do not necessarily indicate a stress response or that an animal is in poor health (Beehner and Bergman, 2017). Although baseline GC levels can estimate the relative fitness of individuals, the relationship varies between species, within populations and at different life stages (Bonier *et al.*, 2009). We found the strongest negative relationship between HCC and marrow lipid content in bison, with little to no relationship in moose or deer. Such differences across taxa are common (Charbonnel *et al.*, 2007; Mislan *et al.*, 2016; Rakotoniaina *et al.*, 2017; Wolf *et al.*, 2018).

Wolves often select prey that are less fit, including young, old, diseased and injured individuals (Huggard, 1993c; Mech and Peterson, 2003; Garrott *et al.*, 2007). Prey that are consumed by wolves may have elevated cortisol and low marrow lipid content, because it is easier for wolves to chase, subdue and kill weaker prey (Smith *et al.*, 2000; Husseman *et al.*, 2003). Vulnerability associated with poor body condition may be particularly important for prey such as bison, because the risk of injury to wolves is higher during predation events with bison compared to other ungulates (Huggard, 1993c; MacNulty, 2002; Tallian *et al.*, 2017). Studies on other predators found that individual kill rate was negatively correlated with prey body condition (Mattisson *et al.*, 2017), indicating that the body condition of prey may help to predict predator success rate.

It is difficult to distinguish if poor body condition causes an increase in circulating GC or if chronic stress causes a decrease in body condition. Animals in poor body condition may exhibit elevated GC concentrations as they must metabolize protein, rather than fat, for energy, resulting in the catabolism of muscle tissue (Cherel *et al.*, 1988; Heath *et al*., 1998). Alternatively, experimentally increasing GC concentrations can adversely affect body condition and lead to muscle wasting (Boonstra, 2004; Rich and Romero, 2005; Charbonnel *et al.*, 2007). Therefore, the effects of chronic stress and body condition on each other are most likely correlated and bidirectional.

Environmental factors that affect cortisol levels and body condition of ungulates include parasites, disease, reproductive output, disturbance, habitat quality, weather, climate, starvation and/or injury. Body condition can be negatively related to parasite infection, although the effect size is variable (Carlsson *et al.*, 2016; Sánchez *et al.*, 2018). We did not examine the effect of parasites on body condition on ungulates in our study area, although there was an outbreak of anthrax due to *Bacillus anthracis* that killed 28 bison in our study area in 2008 (Shury *et al.*, 2009). Human disturbance and a reduction in habitat quality can increase GC concentrations in free-ranging wildlife (Boonstra *et al*., 1998; Creel *et al*., 2002; Ewacha *et al*., 2017). Our study area was located in a relatively remote area of a national park, so sustained human disturbance and habitat degradation was minimal. Hiking trails and roads in our study area, as well as human disturbance (vehicle traffic and hazing off of agricultural land) when animals ranged outside of PANP, may have initiated an acute stress response in individuals (Fortin and Andruskiw, 2003). Severe weather, such as prolonged, cold winters can decrease forage quality for ungulates and result in poor body condition late in the winter (Cederlund *et al*., 1991; Delgiudice *et al*. 2001). We found that deeper snow was correlated with reduced marrow lipid content. Deep snow (>70 cm) can inhibit ungulate movement, restricting access to forage and increasing energetic output, which can lead to malnutrition and starvation (Kelsall, 1969; Sweeney and Sweeney, 1984; Telfer and Kelsall, 1984; Huggard, 1993a). Overall, there are varying effects of seasonality on cortisol and body condition, which may be related to behavioural and environmental differences, such as offspring care and protection, food availability and temperature (Mislan *et al*., 2016; Uetake *et al*., 2018; Heimbürge *et al.*, 2019). Future studies should consider sectioning ungulate hair samples to examine temporal differences in HCC over the growing period.

Sex and age can influence body condition and to a lesser extent chronic stress, although the effects of both vary between and within species (Macbeth *et al.*, 2012; Caslini *et al.*, 2016; Mislan *et al.*, 2016; Di Francesco *et al.*, 2017; Prandi *et al.*, 2018). Sex may interact with other factors, such as reproductive status and sexual dimorphism, to influence body condition and chronic stress levels (Lafferty *et al.*, 2015; Mislan *et al.*, 2016). Male bison had elevated HCC and were in poorer body condition compared to females, but the results were not significant for HCC. GC concentrations may peak during the breeding season of ungulates, when there is an increase in the number of agonistic encounters between males (Reyes *et al.*, 1997; Romero and Butler, 2007; Pavitt *et al.*, 2015), which overlapped with HCC measurements in our study (late summer to autumn).

Our results identify a potential link between body condition and chronic stress, particularly in wolf-killed bison, that suggests wolves may be killing prey in poorer condition. Larger sample sizes may have better represented the potential variation of cortisol and marrow lipids among individuals. In addition, incorporating HCC results from harvested deer and elk may have strengthened the relationship between HCC and mortality source (harvested and wolf-killed) among different species; however, we were lacking this data. Understanding the relationship between body condition/chronic stress and environmental stressors may allow managers to reduce harvest rates during and after years when prey condition is expected to be low. It may also provide information on expected predator success, which can be used to predict predator population dynamics. Finally, the lower HCC observed in human-harvested bison, relative to wolf-killed bison, suggests that hunting may have relatively larger impacts on population dynamics, as hunters appear to remove healthier individuals that are more likely to survive and reproduce successfully.
